# Prospect Theory for Online Financial Trading

**DOI:** 10.1371/journal.pone.0109458

**Published:** 2014-10-15

**Authors:** Yang-Yu Liu, Jose C. Nacher, Tomoshiro Ochiai, Mauro Martino, Yaniv Altshuler

**Affiliations:** 1 Channing Division of Network Medicine, Brigham and Women's Hospital and Harvard Medical School, Boston, Massachusetts, United States of America; 2 Center for Complex Network Research and Departments of Physics, Computer Science and Biology, Northeastern University, Boston, Massachusetts, United States of America; 3 Center for Cancer Systems Biology, Dana-Farber Cancer Institute, Boston, Massachusetts, United States of America; 4 Department of Information Science, Faculty of Science, Toho University, Funabashi, Chiba, Japan; 5 Department of Social Information Studies, Otsuma Women's University, Tama-shi, Tokyo, Japan; 6 Center for Innovation in Visual Analytics, Watson Research Center, IBM, Cambridge, Massachusetts, United States of America; 7 MIT Media Lab, Cambridge, Massachusetts, United States of America; University of Warwick, United Kingdom

## Abstract

Prospect theory is widely viewed as the best available descriptive model of how people evaluate risk in experimental settings. According to prospect theory, people are typically risk-averse with respect to gains and risk-seeking with respect to losses, known as the “reflection effect”. People are much more sensitive to losses than to gains of the same magnitude, a phenomenon called “loss aversion”. Despite of the fact that prospect theory has been well developed in behavioral economics at the theoretical level, there exist very few large-scale empirical studies and most of the previous studies have been undertaken with micro-panel data. Here we analyze over 28.5 million trades made by 81.3 thousand traders of an online financial trading community over 28 months, aiming to explore the large-scale empirical aspect of prospect theory. By analyzing and comparing the behavior of winning and losing trades and traders, we find clear evidence of the reflection effect and the loss aversion phenomenon, which are essential in prospect theory. This work hence demonstrates an unprecedented large-scale empirical evidence of prospect theory, which has immediate implication in financial trading, e.g., developing new trading strategies by minimizing the impact of the reflection effect and the loss aversion phenomenon. Moreover, we introduce three novel behavioral metrics to differentiate winning and losing traders based on their historical trading behavior. This offers us potential opportunities to augment online social trading where traders are allowed to watch and follow the trading activities of others, by predicting potential winners based on their historical trading behavior.

## Introduction

We live life in the “big data” era. Many of our daily activities such as checking emails, making mobile phone calls, posting blogs on social media, shopping with credit cards and making financial trading online, will leave behind our digital traces of various kinds that can be used to analyze our behavior. The sudden influx of data is transforming social sciences at an unprecedented pace [1], [2]. Indeed, we are witnessing the shift of social science research paradigm from interviewing a few dozens of people with crafted survey questionnaire to designing experiments involving millions of subjects on social media [3].

The availability of huge amounts of digital data also prompts us to rethink some fundamental perspectives of complex human behavior. Recent studies have already illustrated the potential that extensive behavioral data sets (e.g., Google trends, Wikipedia usage patterns, and financial news developments) could offer us a better understanding of collective human behavior in financial markets [4]–[7]. In this work we focus on economic decision under risk, a key subject of behavior economics [8]. Successful behavior economic theories acknowledge the complexity of human economic behavior and introduce models that are well grounded in psychological research. For example, prospect theory is viewed as the best available descriptive model of how people evaluate risk [9]–[14]. Prospect theory states that people make decisions based on the potential value of losses and gains rather than the final outcome, and that people evaluate these losses and gains using certain heuristics. Despite the fact that prospect theory offers many remarkable insights and has been studied for more than three decades, there exist very few large-scale empirical research and most of the previous studies have been undertaken with micro-panel data [15]–[21]. Moreover, there are relatively few well-known and broadly accepted applications of prospect theory in economics and finance [14]. The emergence of online social trading platforms and the availability of burgeoning volume of financial transaction data of individuals help us explore the empirical aspect of prospect theory to an unprecedented large-scale. Moreover, analyzing the trading behavior at the individual level offers an excellent opportunity to develop pragmatic financial applications of prospect theory.

By harnessing the wisdom of the crowds to our benefit and gain, social trading has been a revolutionary way to approach financial market investment. Thanks to various Web 2.0 applications, nowadays online traders can rely on trader generated financial content as the major information source for making financial trading decisions. This “data deluge” raises some new questions, answers to which could further deepen our understanding of the complexity of human economic behavior and improve our social trading experience. For example, many social trading platforms allow us to follow top traders, known as gurus or trade leaders, and directly invest our money like they do. The question is then how to identify those top traders. Analyzing their historical trading behavior would be a natural starting point [22].

## Analysis

The financial transaction data used in this work comes from an online social trading platform for foreign exchanges and commodities trading. This trading platform allows traders to take both long and short positions, with a minimal bid of a few dollars as well as leverage up to 400 times. The most important feature of social trading platform is that each trader automatically has all trades uploaded to the platform where trades can be displayed in a number of statistical ways, such as by the amount of profit made. Traders can then set their accounts to copy one or more trades made by any other traders, in which case the social trading platform will automatically execute the trade(s). Accordingly, there are three types of trades: (i) Single (or non-social) trade: Trader A places a normal trade by himself or herself; (ii) Copy trade: Trader A places exactly the same trade as trader B's one single trade; (iii) Mirror trade: Trader A automatically executes trader B's every single trade, i.e., trader A follows exactly trader B's trading activities. Both (ii) and (iii) are hereafter referred to as social trading.

There are about 3 million registered accounts in this online social trading platform. Some of them are practice accounts, i.e., trading with virtual money. Our data are composed of over 28.5 million trades made by 81.3 thousand traders trading with real money from June 2010 to October 2012. There are 31.8% single trades, 0.6% copy trades and 67.6% mirror trades. Apparently, social trading dominates over this trading platform during the time window of our data. It will be desirable to learn how to select the best traders to follow so that we can further improve our social trading experience — a pragmatic motivation of our current work. Quantitatively analyzing trading activities of traders within the framework of behavior economics naturally fits the goal. Ultimately, we would like to be able to predict potential winners based on their historical trading behavior so that we can take full advantage of the social trading paradigm.

## Results

### Social vs. Non-social trade

**Figure 1:**
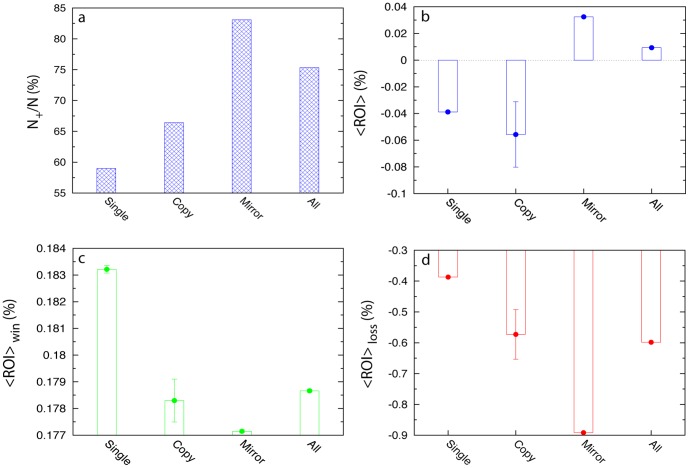
Performance comparison of different types of trades. (**a**) Fraction of positive trades. Mirror trade has the highest fraction of positive trades. (**b**) Mean ROI. Mirror trade is the only trade type that has the positive 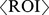. Here error bars mean the standard error of the mean (SEM). (**c**) Mean ROI of positive trades. Mirror trade has the lowest 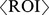 for positive trades. (**d**) Mean ROI of negative trades. Mirror trade has the highest negative 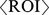 for negative trades.

We first need to demonstrate if social trades really help. In Fig. 1 we compare the fraction of winning trades (*N*_+_/*N*) and return on investment (ROI (%) 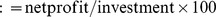) of the three different trade types. We find that all three trade types have more than 50% chance to generate positive net profit (see Fig. 1a). Among them, mirror trade has the highest chance (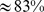), much higher than that of single or copy trade. This indicates that in average social trades (especially mirror trades) indeed help traders win more frequently than non-social trades. Interestingly, not all the trade types have positive average ROI (see Fig. 1b). In fact, only mirror trade has positive average ROI (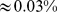), i.e., it generates profit, consistent with previous results [23]. In terms of ROI, social trades do not necessarily perform better than non-social trade. We notice that copy trade even has higher negative ROI than non-social trade, which simply implies that copying someone based on past performance can be dangerous.

Overall, mirror trade outperforms both single and copy trades. Yet, the better performance of mirror trade comes at the price that its winning trades have much lower ROI (≈0.177%) than that of copy and single trades (see Fig. 1c); while its losing trades have significantly higher negative ROI (≈−0.9%) than that of copy and single trades (see Fig. 1d). In other words, mirror trade typically does not generate high profit for winning positions but generate high loss for losing positions. Since mirror trade has very high chance of winning, the average ROI of mirror trade turns out to be positive. This implies that there is still much room to improve our social trading experience.

**Figure 2:**
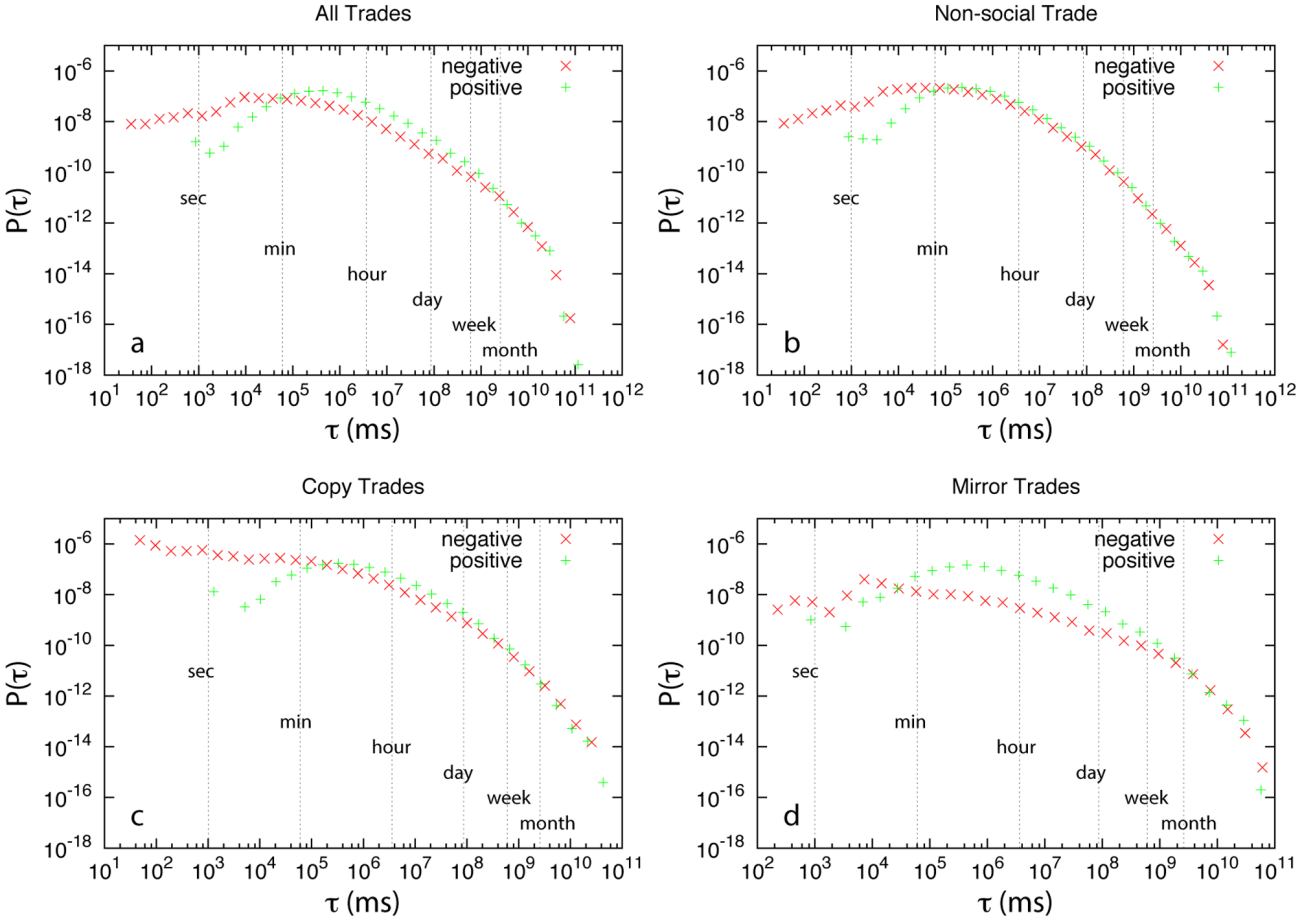
Duration distribution of different trade types. For each trade type, we further distinguish negative and positive trades based on their net profit. The trades with zero net profit are negligible. The duration distributions of negative and positive trades are normalized according to their corresponding occurrence. (**a**) All trades. (**b**) Non-social trades. (**c**) Copy trades. (**d**) Mirror trades.

To further understand the difference between social and non-social trade types, we calculate their duration distributions 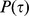 (see Fig. 2). Here the duration 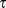 of a trade or position is defined to be its holding time (in unit of millisecond), i.e., 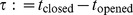 where 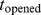 and 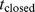 are the position opened and closed time, respectively. Interestingly, 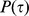 displays similar fat-tailed distribution for all different trade types. There are very few positions that were held for very long time (more than one month). Most of the positions were held for less than half an hour. We also notice that for losing positions, many of them are held for less than one second, while for winning positions they are most likely held for longer than one second. This might be due to the so-called bid-ask spread. The price we can sell (bid) and the price we can buy (ask) is different at each time point. It is almost impossible for traders to overcome the spread within very short holding time interval (e.g., one second) by using online financial trading platforms. For 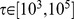, we find that for all different trade types 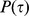 of positive trades is much lower than that of negative trades, i.e., winning probability is much less than 50% in this particular holding time window. Although the duration distributions of different trade types share many similar features, we do observe a noticeable difference in the regime of 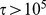, i.e., longer than one minute. We find that for non-social trades with 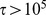, 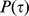 of positive trades is roughly the same as that of negative trades. In other words, if the holding time of a non-social trade is longer than one minute, the winning chance is about 50%. For copy trades with 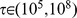, 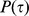 of positive trades is slightly higher than that of negative trades. For mirror trades with 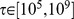, 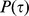 of positive trades is significantly higher than that of negative trades. In other words, if the holding time of a mirror trade is longer than one minute and less than one week, then its winning chance is much higher than 50%.

**Figure 3:**
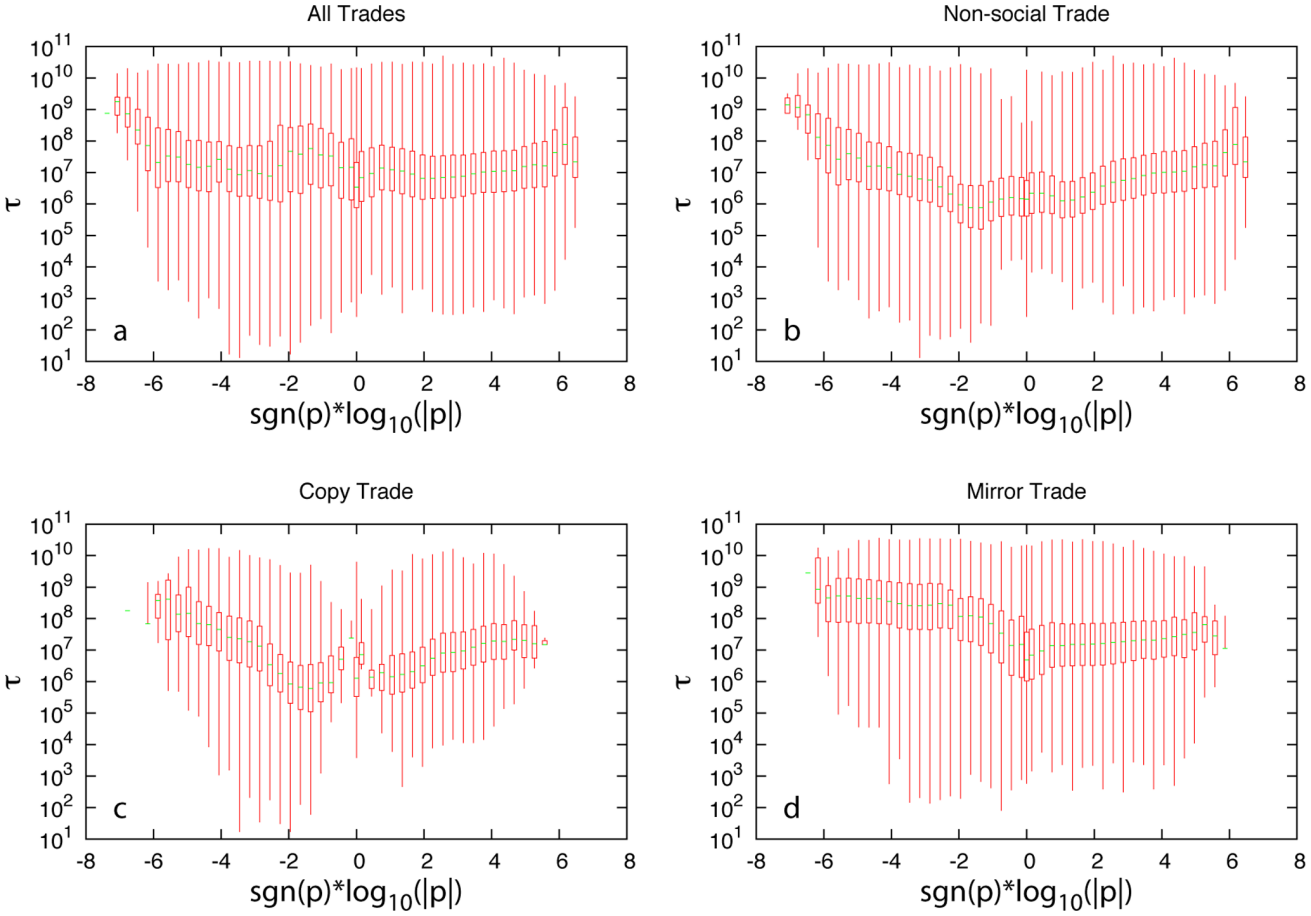
Disposition effect in different trade types. Here, we bin the net profit 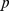 of different trade types in logarithmic bins. (If 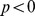, we bin it using 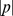.) For the trades contained in each bin, we draw the box-and-whisker plot for their duration (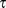), representing the minimum, first quartile, median, third quartiles, and maximum of the data in the bin. (**a**) All trades. (**b**) Non-social trades. (**c**) Copy trades. (**d**) Mirror trades.

We also calculate the trade duration as a function of the net profit for different trade types (see Fig. 3). We draw the box-and-whisker plot of duration (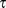) for trades with net profit (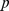) binned logarithmically. (For negative trades 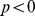, we bin them using 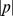.) We denote the median value of durations as 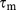. We find that for all trade types 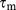 shows asymmetric behavior: 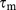 of losing positions with loss 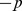 is generally higher than that of winning positions with profit 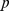. This is a reflection of the so-called “disposition effect”: investors tend to sell financial assets whose price has increased while keeping asserts that have dropped in value [24]–[27]. In other words, investors are less willing to recognize losses, but are more willing to recognize gains. This is a typical irrational behavior that can be partially explained by the “loss aversion” phenomenon and the “reflection effect” in prospect theory [18], [ 28]–[32]. We also notice clear differences between mirror trade and the other two trade types. For both non-social and copy trades 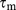 generally increases as 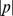 increases in either positive or negative direction, and 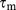 of losing trades increases much faster as 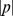 increase than 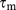 of winning trades increases as 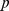 increase. While for mirror trade, 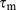 increases very slowly as 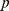 increases for positive positions. For mirror trades with negative 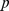, 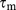 increases initially as 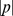 increases, but quickly reaches a plateau. In other words, the disposition effect is lessened in mirror trade. It has been shown that more experienced investors are less affected by the disposition effect [33]. This might explain the good performance of mirror trade.

### Winning vs. Losing traders

To characterize the trading behavior of traders and identify potential trade leaders, we introduce four behavioral metrics: (i) Risk-reward ratio 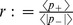, where 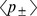 represents the average profit of positive/negative trades made by each trader. 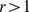 means that traders in average gain more in positive trades than the loss in negative trades. (ii) Win-loss holding time ratio 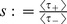, where 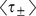 represents the average holding time of positive/negative trades made by each trader. 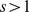 means that traders in average hold positive position longer than negative position. (iii) Win-loss ROI ratio 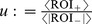, where 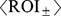 represents the average ROI of positive/negative trades made by each trader. 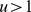 means that traders in average have larger absolute ROI in positive trades than in negative trades. (iv) Winning percentage 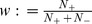, where 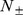 represents the number of positive/negative trades made by each trader. 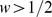 simply means that traders in average have larger chance of winning than losing a trade.

**Figure 4:**
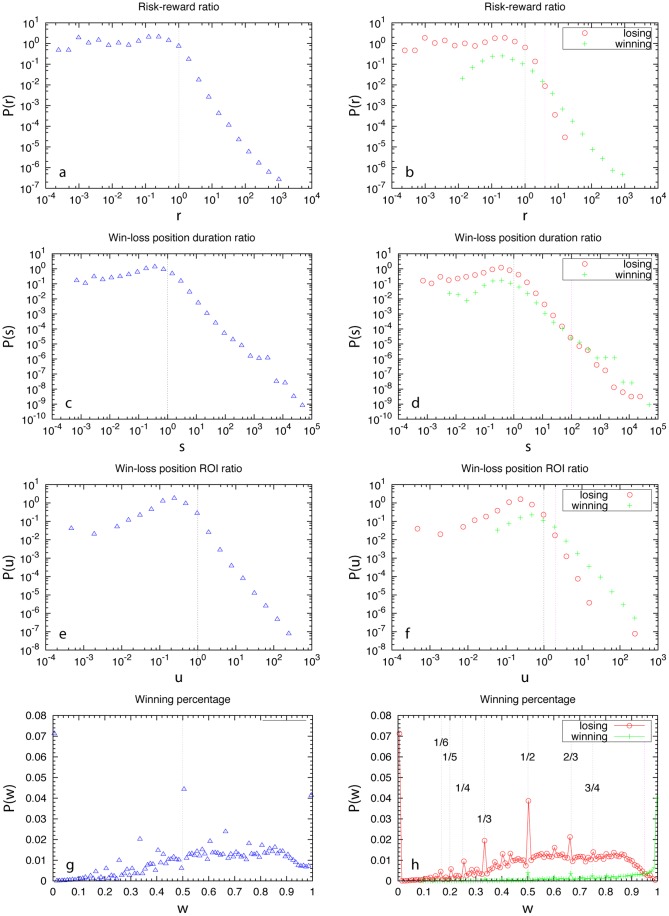
Characterizing winning and losing traders based on historical trading behavior. (**a, b**) Distribution of risk-reward ratio (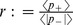). 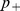 and 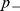 are the profit of winning positions and the loss of losing positions, respectively, of traders. (**c, d**) Distribution of win-loss waiting time ratio (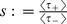). Here 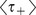 and 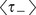 are the average duration time of winning and losing positions, respectively, of traders. (**e, f**) Distribution of win-loss ROI ratio (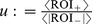). 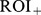 and 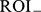 are the ROI of winning and losing positions, respectively, of traders. (**g, h**) Distribution of winning percentage (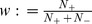) of traders. 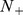 and 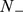 are the number of winning and losing positions, respectively, of traders.

Note that if all traders trade pure randomly without any human emotions we would expect that the distributions of all the four metrics show symmetric behavior around 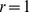, 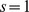, 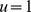 and 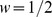, respectively. Yet, in reality traders behave quite differently from random (see Fig. 4). We find the risk-reward distribution 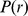 displays strongly asymmetric behavior around 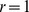 (black dotted line in Fig. 4a). For 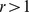, 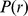 follows a power law over almost 3 decades, which means it is extremely difficult to find traders with very large 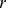; while for 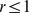, 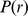 is almost a constant, which means traders with 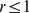 are almost uniformly distributed. By splitting the traders into two groups: winning and losing traders (i.e., traders with final net profit or net loss) and calculating their 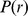 with appropriate normalization based on the fractions of winning and losing traders (14.7% and 85.2%, respectively), we find that the two groups behave in drastically different ways (see Fig. 4b). Winning traders' 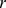-range spans over 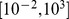; while losing traders' 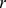-range is given by 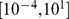. The uniform 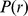 for 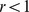 is largely due to losers; while the power-law of 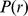 for 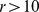 is purely due to winning traders. We also notice that for 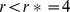 (pink line), 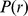 of losing traders is significantly higher than that of winning traders; while for 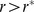, it is in the opposite case.

We find the win-loss duration ratio distribution 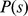 also displays strongly asymmetric behavior around 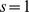 (black dotted line in Fig. 4c). For 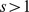, 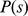 almost follows a power law over 5 decades, which means it is extremely difficult to find traders with very large 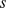; while for 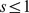, 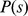 decays very slowly as 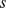 decreases, which means traders with 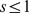 are almost uniformly distributed. This indicates that most traders hold losing positions for a longer time than winning position, a typical disposition effect. Comparing winning and losing traders' 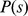 is also interesting (see Fig. 4d). Though their 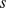-ranges are almost the same, we notice that for 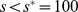 (pink line), 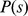 of losers is significantly higher than that of winners; while for 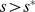, it is in the opposite case.

The win-loss ROI ratio distribution 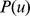 shows a strong peak around 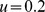 and a strongly asymmetric behavior around 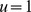 (black dotted line in Fig. 4e). For 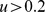, 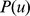 follows a power law over 3 decades, which means it is extremely difficult to find traders with very large 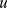. Interestingly, for 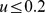, 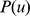 also follows a power-law over almost 2 decades. We find 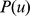
's of winning and losing traders are also very different (see Fig. 4f). Winning traders' 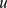-range spans over 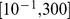; while losing traders' 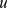-range is given by 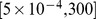. For 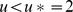 (pink line), 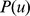 of losing traders is significantly higher than that of winning traders; while for 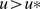, it is in the opposite case. For 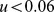, almost all traders are losing traders.

Note that a large portion of traders (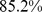) are losing traders with final net loss. The fact that those losing traders typically have 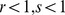 and 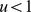 could be explained by the “loss aversion” phenomenon and the “reflection effect” in prospect theory as follows. For positive positions, traders tend to be risk-averse and will close the position quickly to take a small profit or a small ROI. While for losing positions, traders tend to be risk-seeking and reluctant to close the positions as quick as they should. Instead they keep waiting and hoping to recover the loss. If indeed this happens, then they become risk-averse and tend to close the position quickly to take a small profit and result in a small ROI. If unfortunately this does not happen, they waste not only valuable time but also a lot of money, rendering large negative ROI. Thus, the losing traders have to suffer from their irrational trading behavior that can be described by prospect theory.

One may naively consider the winning percentage type of behavioral metrics will help us identify gurus. Here we show it is not the case. Fig. 4g displays the winning percentage distribution 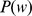, which is asymmetric around 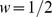. (Note that 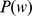 has significant peaks around some rational numbers 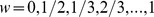, which are due to traders who made very few transactions.) Again, we find winning and losing traders' 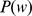 show dramatically different behavior (see Fig. 4h). For 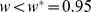, 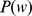 of losing traders is significantly higher than that of winning traders; while for 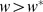 winning traders dominate. The value of 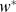 is so high that using it to select trade leaders is almost infeasible. We also notice that for 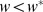, losing traders actually dominate for a wide range of 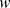. Yet, they are still losing money eventually due to their very low risk-reward ratio 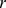. In other words, they win many times with small positive profit, but once they lose they lose a lot.

In principle large values of those metrics do not imply net profit at all. For example, traders with 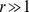 could be simply due to a few trades with very large profit, but many trades with very small loss. Yet, the above analysis yields three characteristic values (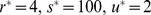) that can be used to statistically predict potential winning traders with high probability. We admit that those characteristic values may slightly depend on the particular dataset or trading platform. We emphasize that the strategy of using characteristic values of novel behavioral metrics to identify potential winning traders should be applicable to general social trading platforms. Furthermore, the existence of characteristic values (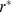, 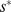, 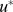) of these behavioral metrics indicates the importance of controlling human emotion to minimize the impact of the reflection effect and the loss aversion phenomenon for better trading performance.

## Discussion

The dynamics of financial trading is governed by individual human decisions, which implies that the trading performance could be significantly improved by understanding better the underlying human behavior. In this work we systematically analyze over 28.5 million trades made by 81.3 thousand traders of an online financial trading platform. By analyzing and comparing the performance of social and non-social trades, winning and losing traders, we find clear evidences of the reflection effect and the loss aversion phenomenon, which are essential in prospect theory of behavior economics. Many losing traders have very small risk-reward ratio (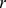), win-loss duration ratio (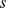), and win-loss ROI ratio (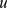), suggest that we should develop new trading strategies by systematically minimizing the impact of the reflection effect and the loss aversion phenomenon, e.g., through intentionally controlling 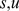 and/or 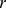 to fight over our human nature and rationalize our trading behavior.

To provide traders many preferences in discovering gurus or trade leaders, social trading platforms rank traders on many different metrics, e.g., popularity (number of followers), profitable weeks, and the personal return of rate calculated from the modified Dietz formula [34], etc. Those different metrics typically yield different ranking lists, which effectively renders choosing the gurus something of an inspirational affair or a delicate trick. Moreover, traders should take into account the risks taken by these gurus in order to obtain the returns that they make. Unfortunately, not all the metrics rank the performance of traders on a risk-adjusted basis. Here we propose three novel behavioral metrics (risk-reward ratio 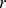, win-loss holding time ratio 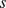, and win-loss ROI ratio 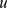), which reveal the essence of prospect theory, the best available descriptive model of how people evaluate risk in behavioral economics. These metrics are defined for each trader by comparing his/her typical behavior in winning and losing trades, and hence are naturally risk-adjusted. Our analysis suggests that these metrics can be used to statistically predict potential winning traders, offering pragmatic opportunities to further improve our social trading experience.
